# Collision of high-resolution wide FOV metalens cameras and vision tasks

**DOI:** 10.1515/nanoph-2024-0547

**Published:** 2025-01-30

**Authors:** Shaoqi Li, Wangzhe Zhou, Yiyi Li, Zhechun Lu, Fen Zhao, Xin He, Xinpeng Jiang, Te Du, Zhaojian Zhang, Yuehua Deng, Shengru Zhou, Hengchang Nong, Yang Yu, Zhenfu Zhang, Yunxin Han, Sha Huang, Jiagui Wu, Huan Chen, Junbo Yang

**Affiliations:** College of Science, National University of Defense Technology, Changsha 410073, China; School of Artificial Intelligence, Chongqing University of Technology, Chongqing 401135, China; School of Physical Science and Technology Southwest University, Chongqing 400715, China

**Keywords:** metalens, large field of view, imaging, computer vision, identification

## Abstract

Metalenses, with their compact form factor and unique optical capabilities, hold tremendous potential for advancing computer vision applications. In this work, we propose a high-resolution, large field-of-view (FOV) metalens intelligent recognition system, combining the latest YOLO framework, aimed at supporting a range of vision tasks. Specifically, we demonstrate its effectiveness in scanning, pose recognition, and object classification. The metalens we designed to achieve a 100° FOV while operating near the diffraction limit, as confirmed by experimental results. Moreover, the metalenses weigh only 0.1 g and occupy a compact volume of 0.04 cm^3^, effectively addressing the bulkiness of conventional lenses and overcoming the limitations of traditional metalens in spatial frequency transmission. This work highlights the transformative potential of metalenses in the field of computer vision, The integration of metalenses with computer vision opens exciting possibilities for next-generation imaging systems, offering both enhanced functionality and unprecedented miniaturization.

## 1 Introduction

The evolution of object detection algorithms, particularly the YOLO (You Only Look Once) framework [1], has significantly advanced the field of computer vision by enabling real-time processing and achieving high accuracy in identifying and classifying objects. As YOLO continues to evolve, integrating its capabilities with innovative optical technologies like metalenses presents exciting opportunities for enhanced visual perception. This combination has the potential to revolutionize applications in robotics, autonomous systems, and smart devices, creating powerful and efficient vision systems that are both lightweight and high-performing.

In recent years, computer vision has experienced remarkable advancements, driven by the demand for real-time analysis and interpretation of visual data. Applications ranging from autonomous vehicles and robotics to augmented reality and surveillance systems depend heavily on efficient and compact imaging solutions. As the capabilities of computer vision algorithms, such as YOLO, continue to advance, the integration of lightweight and high-performance lenses becomes essential. However, traditional high-performance optical elements are bulky and difficult to integrate into compact devices. Metalenses, as emerging light field manipulation elements, offer an excellent alternative to conventional refractive lenses. With their pixel-level wavefront control capabilities, metalens can transform incident wavefronts into ideal spherical waves, achieving perfect imaging [2], [3], [4], [5], [6], [7], [8], [9], [10]. To achieve this, metalens primarily utilizes the following phase modulation mechanisms for wavefront shaping: resonant phase, geometric phase, and propagation phase [11], [12], [13], [14], [15], [16], [17], [18]. The pioneering work on metasurfaces was based on resonant phase control, utilizing V-shaped antennas as resonant elements to induce abrupt phase changes, thereby enabling anomalous refraction. This also introduced the generalized Snell’s law, which provides a theoretical foundation for the design of metalenses [19]. Geometric phase, widely used in broadband achromatic applications [20], [21], [22], [23], is particularly advantageous due to its wavelength-independent nature. The propagation phase, akin to traditional refractive optical elements, modulates the phase of light by controlling its propagation distance through a medium and is commonly employed in the design of polarization-insensitive lenses [21], [24], [25], [26], [27], [28], [29]. Metalens have made remarkable strides in areas such as filtering, polarization conversion, nonlinear optics, optical trapping, and imaging, bringing us closer to the ideal of freely manipulating light fields [30], [31], [32], [33], [34], [35], [36], [37], [38]. Among the various fields of metasurface development, optical imaging is perhaps the most captivating. In earlier designs of metalens imaging systems, the focus was primarily on eliminating chromatic aberration to accurately reproduce the world’s colors [21], [25]. However, in certain application scenarios, color information is not the primary concern – such as in target identification and range detection. In these cases, the imaging field of view, or the extent of the lens’s coverage, takes precedence over color accuracy. Currently, one of the simpler traditional aberration-correcting lenses is the Cooke triplet, composed of three lens elements. However, its imaging quality is limited, and it is challenging to integrate into miniaturized devices. This has shifted attention toward metalens. With their high spatial resolution and a high degree of freedom in wavefront control, metalenses offer a promising solution for large FOV miniaturized imaging systems.

In 2013, Aieta et al. [5] proposed the concept of curved metalenses, theoretically eliminating off-axis aberrations. However, due to fabrication challenges, this design was not experimentally validated. In 2016, Arbabi et al. [39] introduced the concept of double-sided metalenses, where nanostructures are fabricated on both sides of a glass substrate to reduce off-axis aberrations. However, the half FOV was limited to approximately 30°, and fabricating structures on both sides of the substrate posed significant manufacturing challenges. In 2017, Pu et al. [40] proposed a rotationally symmetric large-FOV metalens design called the quadratic metalens, which was easier to fabricate experimentally. However, while this design reduced off-axis aberrations, it introduced on-axis aberrations, leading to axial elongation of the focal point and compromising the lens’s modulation transfer function (MTF). To address this issue, we optimized the quadratic metalens while maintaining rotational symmetry in its phase profile. The objective was to preserve the wide FOV performance of the metalens while achieving near-diffraction-limited MTF. In 2020, Shalaginov et al. [41] employed a similar aperture diaphragm-based metalens design, operating in the long-wave infrared (LWIR) spectrum. However, their unit structures were rectangular and H-shaped, resulting in polarization-sensitive metalens. This polarization sensitivity led to a significant decline in the transmission efficiency of the lens. In the same year, Fan et al. [42] shifted the operating spectrum to the visible range, theoretically demonstrating the feasibility of this design for visible light, though experimental validation was lacking. In 2022, Chen et al. [43] achieved a large field of view by laterally stitching metalenses designed for different incident angles. However, this approach introduced varying camera reference frames for each metalens image, resulting in significant challenges in post-image processing. In 2023, Xie et al. [44] replaced the diaphragm with a filter, leveraging the blue shift of the filter’s transmission peaks at different incident angles to achieve an adaptive diaphragm. However, this approach led to a limited aperture diaphragm and was unfavorable for subsequent achromatic studies. Moreover, none of the aforementioned studies addressed the critical and rapidly developing connection between metalenses and computer vision, nor did they thoroughly validate the feasibility of metalens systems as next-generation intelligent imaging devices. Additionally, numerous other studies have explored similar wide-field metalens designs. Due to space limitations, we will not detail them individually. Instead, we summarize the recent advancements in wide-angle metalens systems and their applications in Table 1, providing a comparison with the work proposed in this study for reference.

**Table 1:** Metalens with wide FOV performance.

Autor	Design feature	FOV	Near-diffraction limit	Wavelength (nm)	Experiments and applications	Comment
Aieta et al. [5]	Curved metalens	20°	False	Undetermined	Without experiments	The curved substrate results in increased manufacturing difficulty
Arbabi et al. [39]	Single-layer double-sided metalens	50°	False	850	Achromatic	The double-sided structure results in increased manufacturing difficulty
Pu et al. [40]	Quadratic metalens	160°	False	490,532,633	Only imaging experiments	Excessive spherical aberration leads to low resolution.
Y.Shalaginov et al. [41]	Metalens with aperture	170°	Ture	5,200,940	Only imaging experiments	The longer wavelength results in limited resolution
Xie et al. [43]	A filter is used as the aperture stop	120°	False	830	Only imaging experiments	The design method cannot be extended to achromatic correction.
Chen et al. [43]	Metalens array	120°	Ture	470	Only imaging experiments	The discrete field of view leads to a degradation in overall imaging quality. The discrete field of view leads to a degradation in overall imaging quality.
Engelberg et al. [45]	Quadratic metalens with aperture	80°	True	850	Only imaging experiments	The distance between the aperture stop and the metalens leads to increased packaging difficulty.
This work	Optimized metalens with aperture	100°	True	633	Imaging experiments, including pose recognition and object classification tasks	By combining the advantages and disadvantages of previous work, the metalenses exhibit excellent overall performance.

Overall, in this work, we address the limitations of traditional optical lens systems, which are often bulky, as well as the insufficient resolution of conventional wide-field metalenses. By overcoming the fabrication challenges associated with visible-spectrum metalenses, we bridge the gap in wide-field, near-diffraction-limited metalenses for the visible range and demonstrate their exceptional performance in various visual tasks. Specifically, we designed a single-piece metalens operating at a visible wavelength of 633 nm, achieving near-diffraction-limited performance over a wide angular field of view of 100°.

Compared to conventional short-focus wide-field lenses, we opted for a longer focal length of approximately 2 mm, with a numerical aperture of around 0.7. This lens design successfully accommodates various visual tasks at different distances, including near-range scanning, far-field pose recognition, and object classification at intermediate distances. In the experiments, we effectively utilized the metalens camera to accomplish these tasks. This successful integration of metalenses with computer vision not only demonstrates their potential in various applications but also opens exciting avenues for future advancements in imaging systems.

## 2 Results

### 2.1 Design

In the early stages of metalens development, designs mainly primarily plasmonic or metallic materials. However, due to their significant energy loss, the focus gradually shifted towards all-dielectric metalens. Dielectric materials can achieve high transmission and low loss by overlapping electric and magnetic dipole resonances. By carefully selecting the unit cell structures to align their resonant modes, the Kerker effect can be achieved, enabling a full 2π phase coverage. Currently, commonly used materials in the visible spectrum include TiO_2_, GaN, and Si_3_N_4_.

**Figure 1:**
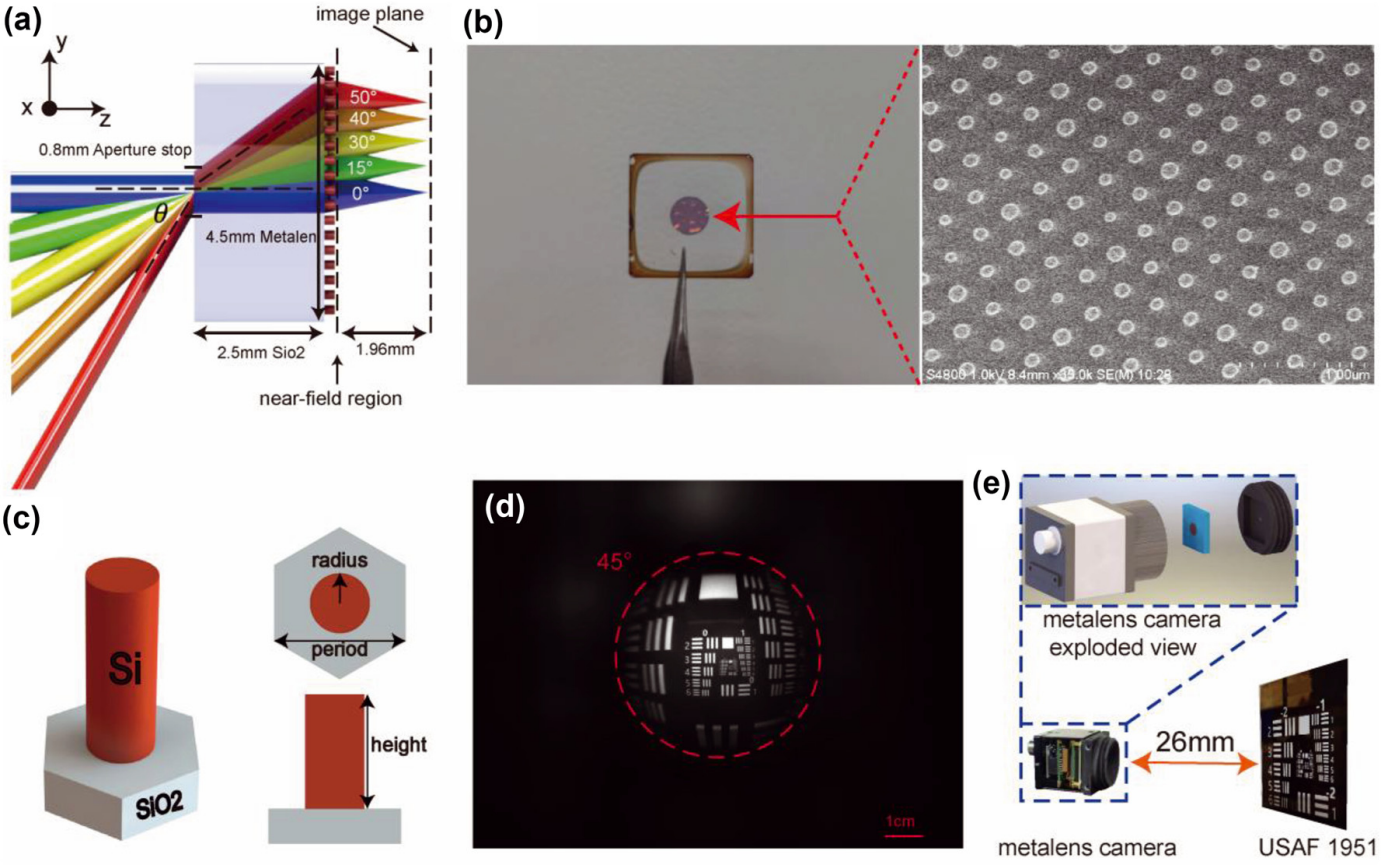
Metalens camera structure and imaging experiments. (a) Schematic of the aperture-stop metalens. (The different colors of the light rays represent only the different incident angles and are unrelated to the wavelength. The corresponding incident angles are explicitly labeled in the figure.) (b) The fabricated metalens sample and its corresponding localized electron microscopy images. (c) Schematic diagram of the unit structure. (d) The imaging effect of metalenses on resolution charts (marked in red as the half field of view). (e) Resolution chart imaging experimental setup diagram.

We have created a metalens that operates at 633 nm using silicon (Si) unit structures. These unit structures have an average transmittance of over 80 % and can achieve a phase coverage of 0-2π, allowing for precise control of wavefronts. In terms of material selection, we chose Si instead of the lower-loss TiO_2_ for the following reason: we conducted a parameter scan on TiO_2_ cylinders with periods ranging from 200 to 310 nm and heights from 200 nm to 500 nm but were unable to find a TiO_2_ structure that could cover the 0-2π phase range. While increasing the height of TiO_2_ might satisfy this phase condition, a higher aspect ratio would increase the fabrication difficulty with our current processing capabilities. We believe that the inability to achieve the 0-2π phase coverage is due to the relatively low refractive index of TiO_2_ at the 633 nm wavelength. Therefore, we selected Si and successfully identified a suitable unit structure. As for the loss in Si, although Si has higher losses compared to TiO_2_ at 633 nm, this loss is acceptable. Significant loss in Si typically occurs at wavelengths below 500 nm. The simulation data for the unit structures were obtained using the FDTD. The metalens were built with an eight-level phase design, where we selected unit structures that maintained high transmittance while covering the full 0-2π phase range. To avoid higher-order diffraction, the period of the unit structures was kept smaller than the wavelength, and to satisfy the sampling theorem, the period needed to meet the condition $p<\frac{\lambda }{2\text{NA}}$ . In practical fabrication, however, the period could not be too small due to manufacturing constraints. After considering these factors, we selected cylindrical unit structures with a period of 347 nm and a height of 378 nm, as shown in Figure 1c (transmittance and phase distribution of the selected eight unit structures are provided in Supplementary Material S1).

In Figure 1b, you can see the metalens sample that has been fabricated, with a close-up SEM image of the metalens displayed on the right side. In the field of metalens design, quadratic metalens is a well-established approach for achieving wide FOV performance. However, this method often overlooks the Modulation Transfer Function (MTF), a critical metric in optical systems that gauges imaging quality. To address this issue, our metalens design not only focuses on wide FOV but also emphasizes achieving near-diffraction-limited MTF. Theoretically, this design can achieve near-diffraction-limited performance over a 100° FOV at a wavelength of 633 nm. The design process for the metalens primarily involves using ZEMAX to optimize the surface phase. The optimized phase distribution of the metalens is described by Equation (1), where *a*_*i*_ are the polynomial coefficients. The main objective of the optimization is to enhance the lens’s MTF.

(1) $$\begin{array}{c} \psi \left(r\right)=-\overset{n}{\underset{i=1}{\sum }}a_{i}r^{2i} \end{array}$$

The metalenes operate at a wavelength of 633 nm within the visible spectrum. Its structure consists of three key components: an aperture stop at the front, a glass substrate, and the metalens itself. The aperture stop allows for precise control of the metalens’s local regions in response to incident light at different angles, enabling control over light at varying incident angles. Significantly reducing the phase control burden compared to traditional single-layer metalens without an aperture stop. In optical systems, the effect of the aperture stop on image quality is complex. Simply reducing the aperture size does not necessarily enhance imaging quality; an excessively small aperture can increase diffraction effects, reduce light throughput, and block high-frequency information, ultimately degrading image quality. After careful optimization, an aperture stop size of 0.8 mm was selected for this work, with a lens radius of 2.25 mm and a focal length of 1.96 mm. The structural schematic is shown in Figure 1a.

The integrated metalens camera primarily consists of a metalens barrel and a sensor. The metalens barrel is manufactured using 3D printing technology, with a 0.8 mm aperture stop at the front. Figure 1d shows the imaging performance of the metalens camera on the resolution test chart. The dashed area in Figure 1e shows the detailed exploded view of the metalens camera. In the resolution test experiment, we used an LED with a central wavelength of 633 nm as the light source. To prevent overexposure, a diffuser was placed behind the resolution chart to act as a secondary light source. The integrated metalens camera was positioned 26 mm in front of the LBTEK RTS3AB-N resolution chart. Transmission test chart. The camera successfully resolved the Group 2, Element 5 stripes, which correspond to a line width of approximately 0.0787 mm, sufficient for most vision scenarios. It is worth noting that in the resolution tests, the effective half field of view of the metalens-integrated camera reached only 45°, slightly lower than the designed 50°. This discrepancy is primarily attributed to vignetting caused by the thickness of the aperture diaphragm. The 3D-printed diaphragm inherently has a thickness limitation, introducing an additional vignetting effect in the metalens system. To mitigate this issue, the thickness of the aperture diaphragm can be effectively reduced by coating a reflective film on the back surface of the metalens to serve as the diaphragm or by using a metal-based aperture diaphragm. Further details and data supporting this discussion can be found in Supplementary Material S5.

### 2.2 Strehl ratio and MTF

**Figure 2:**
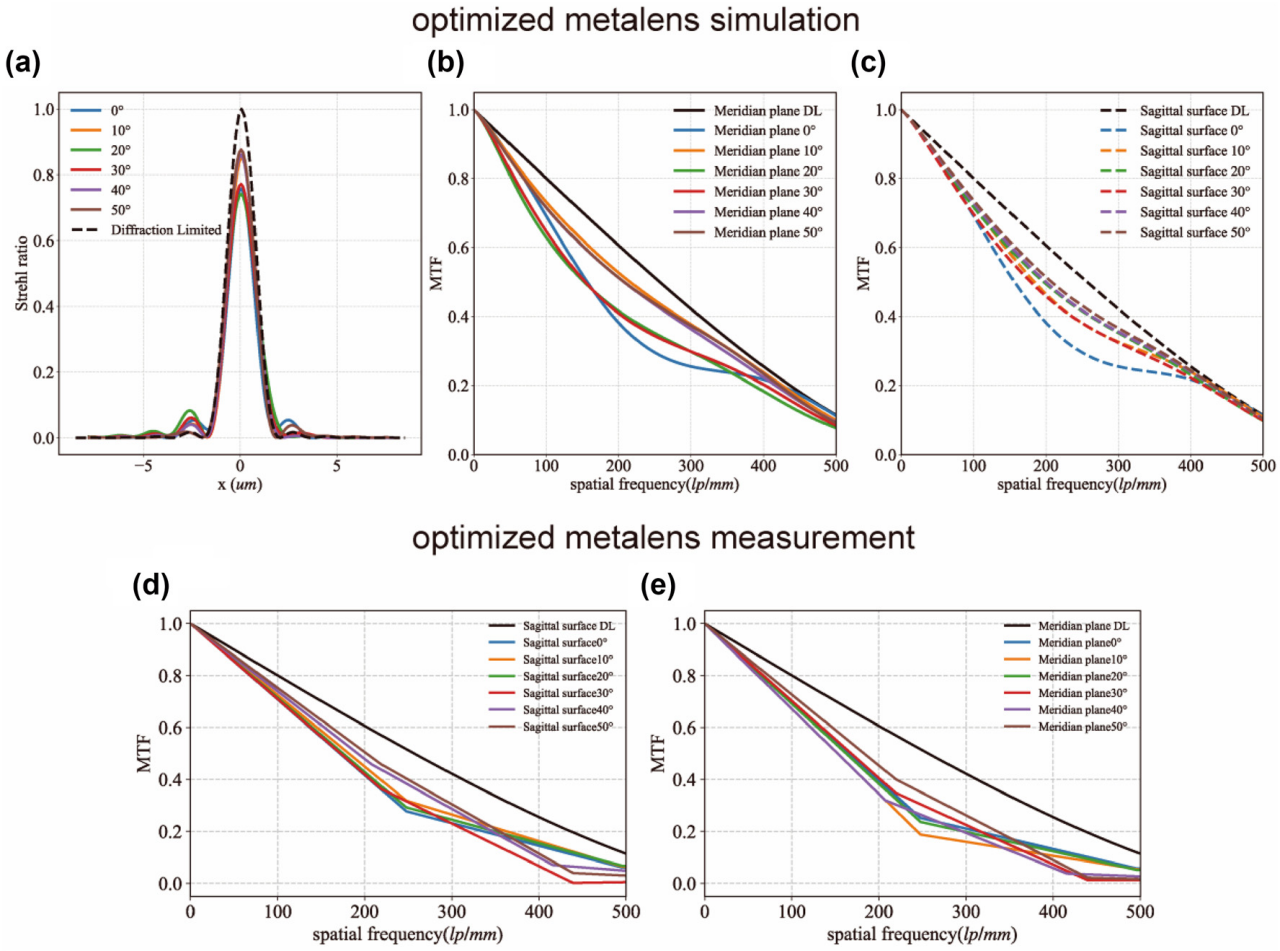
Simulation and experimental results of metalens performance. (a–c) Simulated Strehl ratio and simulated MTF in the meridional and sagittal planes for the optimized metalens. (d) Experimentally measured MTF data in the sagittal plane for the optimized metalens. (e) Experimentally measured MTF data in the meridional plane for the optimized metalens.

The Strehl ratio is commonly used to assess the optical imaging quality of a system. It is defined as the ratio of the peak intensity of the system’s point spread function (PSF) to the peak intensity of an ideal, aberration-free PSF. In imaging lens design, a Strehl ratio of one indicates perfect imaging. However, this ideal scenario is nearly impossible to achieve in practice. During the lens design process, an optical element is generally considered diffraction-limited if its Strehl ratio exceeds 0.8. In this study, the proposed near-diffraction-limited wide-field metalens features a theoretical half FOV of 50°, which corresponds to a full FOV of 100°. The optimized metalenses achieved an average Strehl ratio of 0.75 over an incidence angle range of 0–50°, just 0.05 below the diffraction-limited standard of 0.8, supporting its classification as a near-diffraction-limited optical system. Detailed data are presented in Figure 2(a). In contrast, conventional quadratic metalens have an average Strehl ratio of only approximately 0.35 over the same incidence angle range. The optimized metalens, not only correct off-axis coma but also minimizes on-axis spherical aberration, resulting in significantly improved imaging performance.

The MTF is a crucial parameter for evaluating the imaging quality of an optical system because it quantifies the system’s ability to transmit different spatial frequencies. To validate the MTF data for the optimized metalens, we performed a discrete Fourier transform of the PSF from the metalens optical system during the experiment to obtain the MTF data. Figure 2d shows the experimentally measured MTF in the sagittal direction for the optimized metalens, while Figure 2e shows the MTF measured in the meridional direction. The experimental MTF closely aligned with the theoretically predicted MTF, with minor discrepancies likely attributable to several factors:

1. Light Source Bandwidth: Although the metalens system was designed to operate at a monochromatic of 633 nm, the laser used in the testing apparatus inherently has a certain bandwidth. This spectral width can introduce errors into the measurement system.
2. Focal Spot Magnification System: The focal spot produced by the metalens optical system is comparable in size to the detector’s pixel size, typically on the order of micrometers. Without a magnification system in the focal plane of the metalens, observing the entire morphology of the focal spot using a detector. However, as the magnification system is not a perfect imaging system, some frequency components are lost when transmitting the PSF of the metalens system, leading to a degraded MTF measurement.
3. Electronic Noise: Noise in the electronic equipment introduces random intensity fluctuations in the captured image, which can obscure or distort the true signal.

These factors collectively contribute to the slight deviations observed between the measured and predicted MTFs.

### 2.3 Wavefront

We further examined the imaging performance of the optimized metalens from the perspective of the emergent wavefronts. The overall diameter of the metalens was 4.5 mm, with an aperture stop of 0.8 mm at the front. In a wide-field imaging system without an aperture stop, it is challenging to maintain consistency between the entire emergent wavefront and the ideal spherical wavefront. However, by introducing an aperture stop, it is possible to achieve near-ideal spherical wavefronts in localized regions.

**Figure 3:**
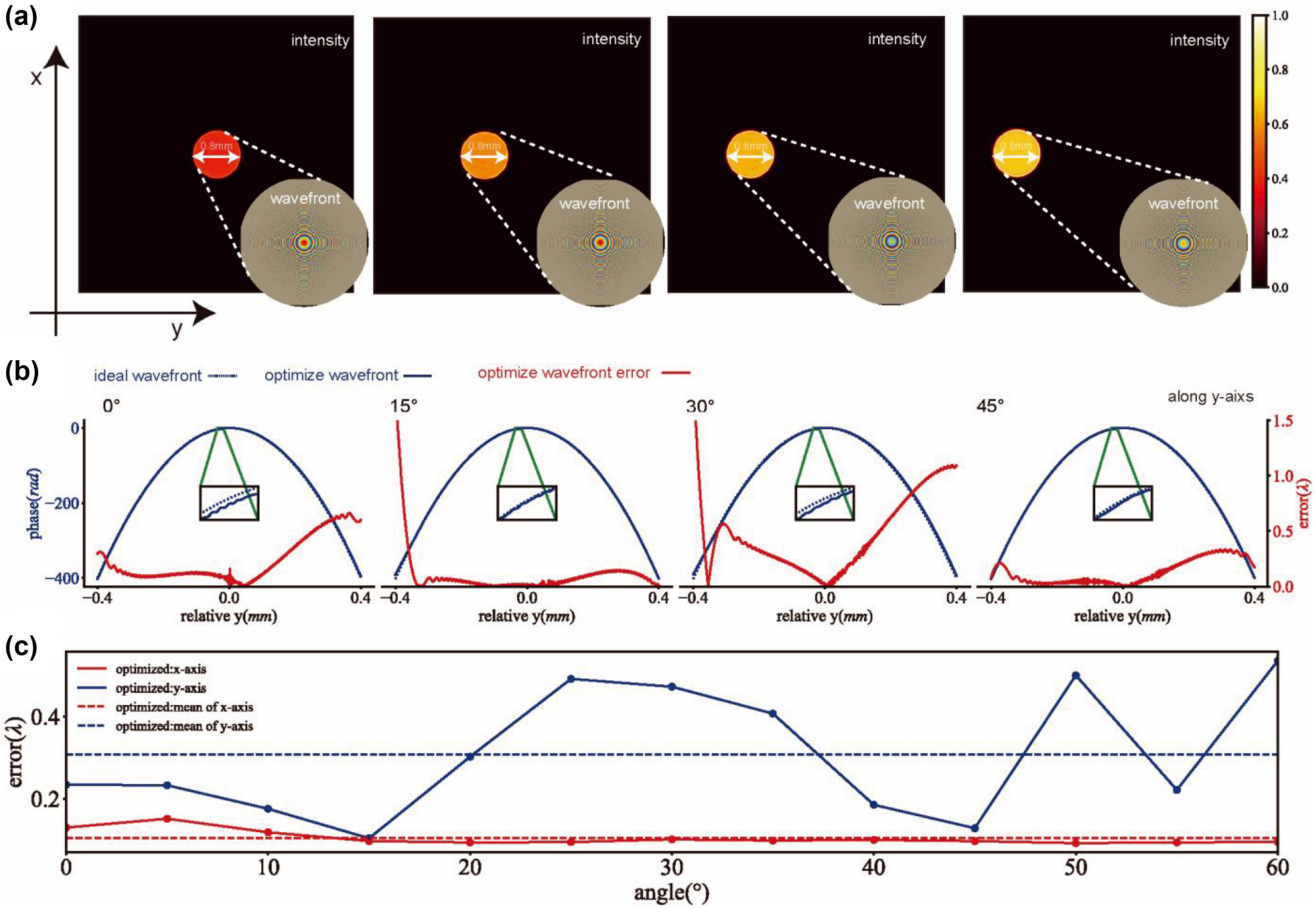
Metalens wavefront calculation. (a) Two-dimensional wavefront and intensity distribution in the near-field region. (b) One-dimensional wavefront phase and wavefront error along the *y*-axis. The solid blue line represents the emergent wavefront of the optimized metalens, while the short blue dashed line represents the ideal wavefront. The solid red line shows the wavefront error of the optimized metalens relative to the ideal wavefront. (c) Solid lines represent the average wavefront error in the *x* or *y* direction for different incident angles, while dashed lines represent the overall average wavefront error in the *x* or *y* direction across all angles. For instance, the overall average value of the green solid line corresponds to the value of the green dashed line.

In optical design, it is generally accepted that the closer the emergent wavefront is to an ideal spherical wavefront, the better is imaging quality of the lens. Based on this concept, we defined a wavefront error function (see Supplementary Material S2) and simulated the near-field wavefront and intensity distribution of the metalens when the aperture stop is placed in close contact with it, as shown in Figure 3a.

Figure 3a shows the wavefront phase and intensity along the *y*-axis for incident angles ranging from 0° to 45° (as the angle increased along the *y*-axis, as indicated in Figure 1a). Figure 3b presents a 2D view of the wavefront phase and intensity. As the incident angle increases, the primary energy distribution in the near-field region shifts upward along the *y*-axis. However, the primary energy of the emergent wavefront remains concentrated within the 0.8 mm region, corresponding to the size of the aperture stop. Thus, within the main energy distribution area, the emergent wavefront of the optimized phase metalens is largely consistent with the ideal spherical wave, demonstrating the excellent imaging performance of the optimized metalens.

Subsequently, we calculated the wavefront error relative to the ideal wavefront for the optimized metalens at incident angles ranging from 0° to 45°(Figure 3c).

### 2.4 PSF

**Figure 4:**
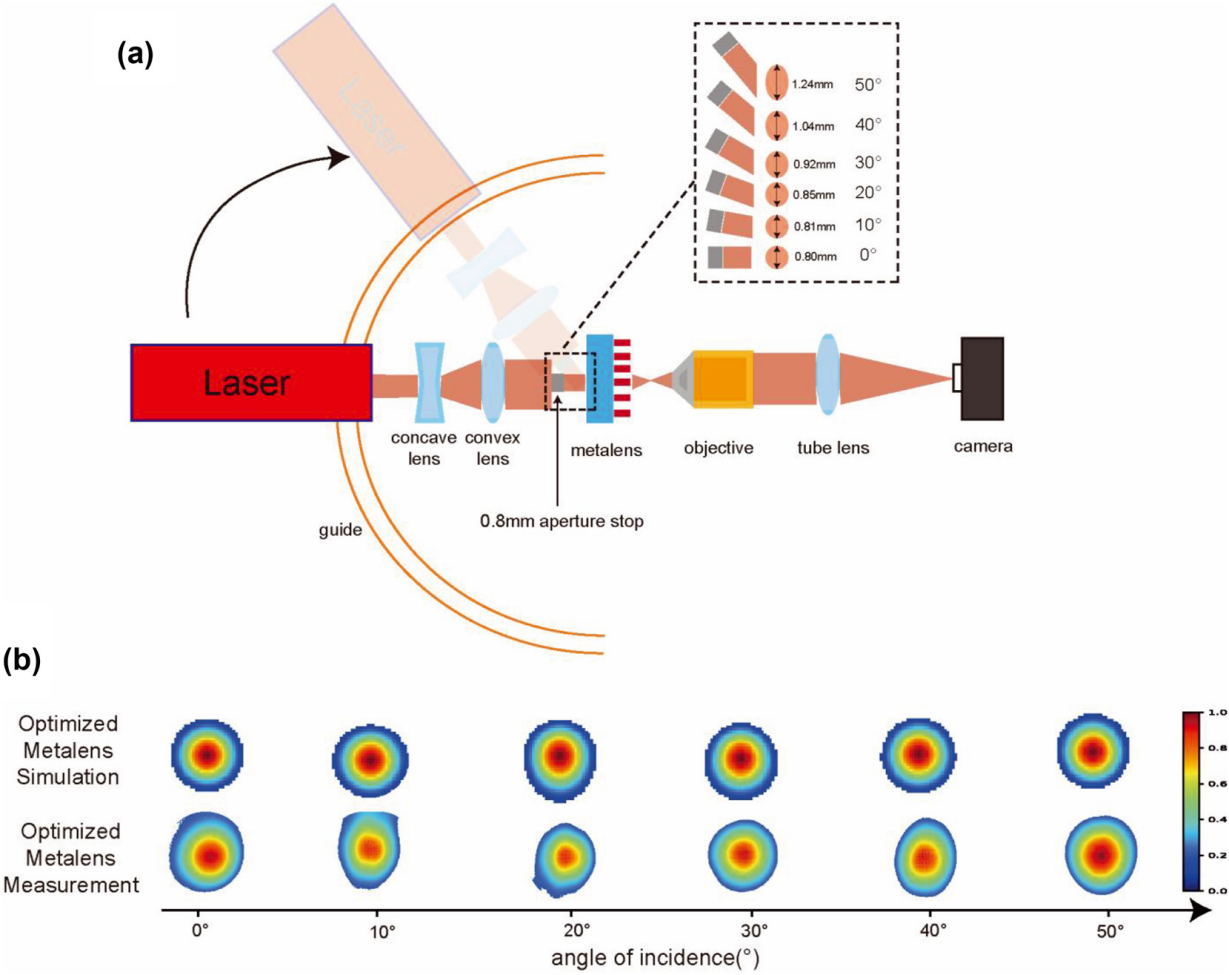
PSF simulation and testingpsfsimula. (a) Schematic of the setup for measuring the PSF of the metalens. (b) Simulated and experimentally measured PSFs for the optimized metalens under incident angles ranging from 0° to 50°.

The imaging process of an optical system can be considered as the integration of an ideal, aberration-free image with the optical system’s PSF. Therefore, the PSF directly reflects the imaging performance of the system. As a comprehensive evaluation metric, As a comprehensive evaluation metric, observing the PSF shape provides a basic assessment of the optical system’s quality. For a wide-field optical system that is free of off-axis aberrations, the PSF should ideally maintain a consistent circular shape across different field angles. Figure 4b, shows the simulated PSFs for the optimized phase designs, and the experimentally measured PSF for the optimized metalens, all under incident angles ranging from 0° to 45°. To enhance clarity when plotting the simulated PSFs, we excluded regions with energy intensity below 0.1 times the peak value. The complete PSF measured in the experiment can be found in Supplementary Material S7. The optimized phase design exhibits almost no noticeable sidelobes. In most large FOV metalenses, strong sidelobes often occur across the field due to on-axis spherical aberrations introduced while compensating for off-axis aberrations. This arises when the lens edges fail to provide a sufficient phase gradient, causing light from various regions to converge at different focal planes. By introducing an aperture stop, we can effectively truncate marginal rays carrying significant aberrations from the lens edges. This approach reduces the on-axis spherical aberration and, improves overall optical performance.

In subsequent experiments, we measured the PSF of the optimized metalens, and the results largely aligned with the theoretical predictions. During the measurement process, accurately aligning the aperture stop with the metalens proved challenging. To address this, we used a 0.8 mm aperture stop in the light source system to produce a parallel beam with a 0.8 mm diameter, simulating the ideal aperture stop condition. This method eliminated alignment errors but introduced a diffraction distance between the aperture stop and the metalens. At larger incident angles, this diffraction distance caused the beam width to expand, therefore, the light spot on the rear surface of the metalens changed from circular to elliptical (dashed areas in Figure 4a), which slightly degraded the metalens’ measured data. The experimental results showed that this beam expansion had minimal impact on the metalens’s performance. This indicates that our optimized metalens is tolerant to errors in the aperture size., and this will not affect the imaging quality of the integrated metalens camera. In such a camera, the aperture stop is housed within the lens barrel, with virtually no distance between the aperture stop and metalens.

The experimental setup for measuring the optimized metalens (Figure 4a), primarily consisted of a 633 nm laser, beam expander system, metalens, magnification system, 0.8 mm aperture stop, and camera. The laser, beam expander system, and 0.8 mm aperture stop were mounted on a rail, allowing the entire assembly to move along the track to measure the metalens data at different incident angles (Supplementary Material S3 for details of the actual experimental equipment). The dashed circles in Figure 4a highlight the beam broadening effect caused by the distance between the aperture stop and metalens.

### 2.5 Vision experiments

In the vision experiments, the captured images still exhibited some blurring at the edges, mainly due to the misalignment of the aperture stop and the vignetting caused by the thickness of the aperture stop. Since a mechanical aperture was used, placing the metalens substrate into the 3D-printed housing may result in a misalignment of the aperture stop with the metalens’ center, caused by unevenness in the housing or deviations in the printing process. This misalignment can cause the main energy region of the outgoing wavefront to fall on the unoptimized areas of the metalens, leading to significant aberrations (a discussion on aperture misalignment can be found in Supplementary Material S4, and a discussion on vignetting caused by the thickness of the aperture stop is included in Supplementary Material S5). This issue can be effectively resolved by coating the opposite side of the metalens substrate with a reflective film to replace the mechanical aperture stop. Reflective films typically have negligible thickness compared to mechanical aperture stops, which helps reduce vignetting effects, and aligning the coating area with the metalens during fabrication is easier, ensuring clearer imaging performance at the edges. Overall, due to assembly limitations, the metalenses camera was unable to fully showcase the optimized metalens’ complete imaging performance.

**Figure 5:**
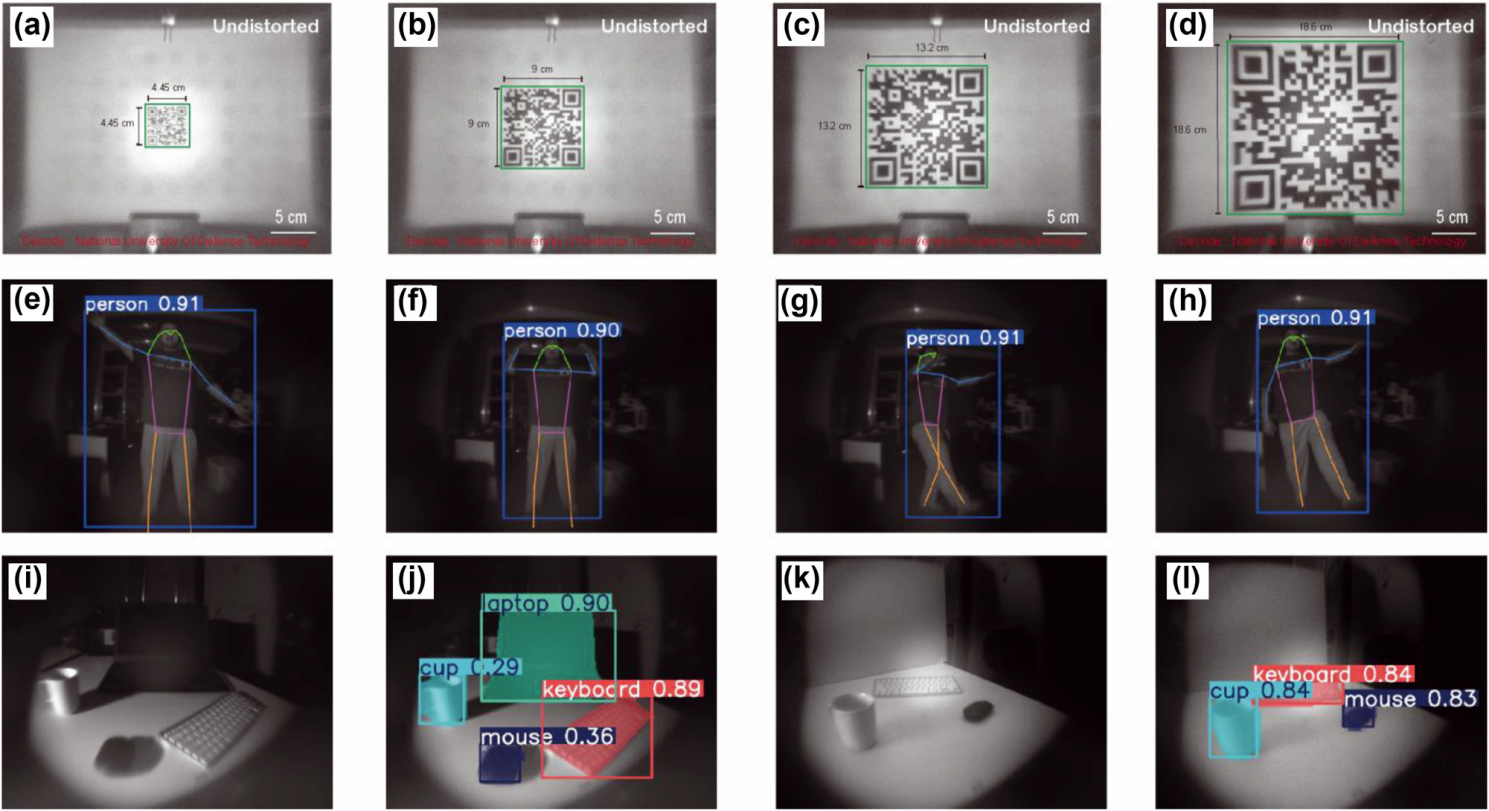
Target recognition and detection. (a–d) QR code and barcode scanning experiments were conducted using the integrated metalens camera, with barcode sizes indicated in black text. The barcode areas identified by the program are highlighted in green boxes, and the decoded content is displayed in red text below. (e–h) Pose recognition experiment. Human body poses are represented using colored line diagrams. (i–l) Object recognition experiment. Different objects are enclosed in boxes of various colors, with the object names labeled above each box.

First, we tested the scanning capabilities of the integrated metalens camera by printing QR codes of different sizes, as shown in Figure 5a–d. We encoded and “National University of Defense Technology” into barcodes and QR codes, placing the QR codes 12 cm away from the integrated metalens camera for imaging experiments. To address the image distortion, we calibrated the metalens using a checkerboard pattern to obtain the intrinsic parameters and distortion coefficients of the integrated metalens camera, which were then used for distortion correction (details of the calibration process can be found in Supplementary Material S6). The experimental results demonstrated that the integrated metalens camera successfully decoded both barcodes and QR codes of various sizes.

Subsequently, we conducted pose recognition experiments using the metalens camera. Pose estimation is a task that involves identifying the locations of specific points in an image, which are commonly referred to as key points. These key points represent various parts of an object. We performed pose recognition using the YOLO11 model, which was specifically trained on the COCO8-pose dataset to satisfy the demands of pose estimation tasks. We stood at a distance of over 3 m from the metalens camera, posed it in different positions, and took pictures with the metalens camera. These images were then fed into a deep-learning model for pose recognition. Figure 5e–h display the results of the pose recognition, which indicate that the metalens pose recognition system effectively identified the key points on the human body and accurately constructed the human pose from these key points.

Finally, we demonstrated the metalens classification recognition system, which also utilized the YOLO11 framework and was trained on the COCO dataset to address classification tasks. We positioned the metalens classification recognition system approximately 1 m away from the scene to capture images of objects on a tabletop. The imaging results of the metalens system are shown in Figure 5i and k, with the corresponding recognition outcomes displayed in Figure 5j and l. When the lighting conditions are poor, resulting in shadows on the objects, the deep learning model exhibits some hesitation during recognition and classification, as seen with the cup and mouse in Figure 5i. However, adjusting the light source to minimize shadows significantly improves the model’s discrimination accuracy. This experiment highlights a key area for optimization in current deep learning frameworks: enhancing the model’s adaptability to variations in lighting, such as exposure changes or shadows, in visual tasks.

In summary, obtaining high-quality images of a scene is a fundamental prerequisite for performing visual tasks. Emerging metalenses can effectively provide high-quality images for computational vision frameworks. This study connects the metalens camera system with computer vision, showcasing its potential as a next-generation imaging technology while also driving computer vision toward broader applications. These two systems complement each other synergistically. The YOLO framework is a classic AI architecture that applies to general imaging systems. However, in certain specialized devices, the presence of metalenses becomes particularly important. For example, in miniature devices such as micro-robots, which are currently receiving significant attention, traditional imaging systems require bulky lens structures to achieve good imaging quality. In this study, the metalenses that actively modulated the optical wavefront had a volume of only 0.04 cm^3^ and a thickness of 0.25 cm. Traditional lenses of the same size are generally unable to achieve the imaging performances presented in this study. We demonstrated that such a thin and lightweight lens can provide high-quality images required for computational imaging. This study provides a promising solution for rapidly developing miniaturized intelligent imaging devices and counters some of the recent doubts surrounding metalenses.

## 3 Conclusions

As computer vision applications expand in areas such as autonomous driving, robotics, augmented reality, and smartphones, traditional imaging systems face limitations, particularly when it comes to achieving higher resolution, wider fields of view, and more compact designs. Metalenses, with their ability to manipulate light at the subwavelength scale and correct for various aberrations, present an attractive solution to these challenges. In this work, we designed high-resolution, wide-field metalens specifically tailored for diverse visual scenarios. For the first time, we utilized the YOLO11 framework to construct an intelligent metalens camera system. Experimental results demonstrate that this system effectively handles close-range scanning tasks, medium-range classification, and recognition tasks, and even achieves pose recognition at relatively longer distances. Specifically, we optimized the metalens surface phase distribution for the visible light wavelength of 633 nm and designed it with a moderate numerical aperture. Additionally, we incorporated an aperture stop in front of the lens to further refine the optical performance by controlling marginal rays and reducing aberrations. This approach enhances the versatility and applicability of the metalens in diverse imaging tasks. We employed silicon (Si) as the material for the fundamental unit structure of the metalens. The refractive index has a real part of 3.8812 and an imaginary part of 0.0189 at 633 nm. By introducing an aperture stop, we achieved perfect imaging with the metalens. Theoretically, our optimized metalens can achieve near-diffraction-limit imaging with a 100° wide field of view, and the average Strehl ratio of the optimized metalens is about 0.75. The metalens we designed have an effective imaging area that weighs only 0.1 g and has a volume of approximately 0.04 cm^3^, effectively approaching the shortcomings of bulky traditional equipment and the poor spatial frequency transfer capability of traditional single-layer metalens.

Due to limitations in experimental packaging technology, we successfully validated high-resolution imaging with a 90° field of view in our experiments. At a distance of 26 mm, our metalens can resolve line widths as small as 0.0787 mm, meeting the requirements for most scanning scenarios. Further optimization of the packaging process could lead to even better imaging performance. Subsequently, we constructed an intelligent visual system for the metalens camera based on the YOLO11 framework, conducting experiments focused on scanning, pose recognition, and classification recognition. First, we placed a QR code approximately 12 cm away from the metalens recognition system for scanning and identification. Next, we used the system to recognize various objects located about 1 m away on a desktop. Finally, we employed the system for pose recognition of a human subject at approximately 3 m in distance. The experimental results were highly promising, demonstrating the system’s effectiveness in various visual tasks.

We anticipate further integration of advanced algorithms and improved metalens designs, which could enhance performance and expand the capabilities of the system in more complex and dynamic environments. This integration may lead to significant advancements in applications such as robotics, smart surveillance, and augmented reality, paving the way for smarter and more efficient visual systems.

## 4 Methods

Due to the high computational requirements of traditional FDTD methods when calculating large-aperture metalenses, we considered the computational cost and treated the metalens as a purely phase-modulating element when calculating the outgoing wavefront. We utilized the actual fabrication sampling rate and employed the angular spectrum diffraction algorithm to compute the outgoing wavefront of the metalens. While this algorithm may introduce some errors in cases of significant coupling between unit structures, we first verified that the chosen unit structure does not exhibit substantial coupling by using FDTD to calculate the performance of a small-aperture metalens with the same unit structure. Based on this validation, we then proceeded to use the angular spectrum diffraction algorithm to evaluate the wavefront performance of the large-aperture metalens.

The fabrication of metalens primarily involves the following steps: 1. Grow a 378 nm-thick layer of silicon on a quartz substrate using PECVD (Plasma-Enhanced Chemical Vapor Deposition). 2. Spin-coat the substrate with PMMA (Polymethyl methacrylate) photoresist, and pattern it using electron beam lithography. 3. Deposit a 30-nm-thick layer of chromium by electron beam evaporation. 4. Use acetone, ethanol, and deionized water to ultrasonically strip the chromium, obtaining a hard chromium mask. 5. Perform silicon etching to a depth of 378 nm using ICP (Inductively Coupled Plasma) etching. 6. Remove the residual chromium using a chromium etchant solution. 7. Clean the substrate with acetone, ethanol, and deionized water to remove organic residues.

## Supplementary Material

Supplementary Material Details
