# Supplementary material for: Collision of high-resolution wide FOV metalens cameras and vision tasks
Source: Nanophotonics. 2025 Jan 30;14(3):315–26. doi: 10.1515/nanoph-2024-0547 (PMC11831395; doi:10.1515/nanoph-2024-0547)
Supplement: Supplementary file 1 — Supplementary Material Details [file j_nanoph-2024-0547_suppl_001.docx]

Supporting Information For:

Collision of High-Resolution, Wide Field-of-View Metalens Cameras and Vision Tasks

Shaoqi Li^1^, Wangzhe Zhou^1^, Yiyi Li^1^, Zhechun Lu^1^, FenZhao^1,2^, Xin He^1^, Xinpeng Jiang^1^, DuTe^1^, Zhaojian Zhang^1^, Yuehua Deng^1^, Shengru Zhou^1^, Hengchang Nong^1^, Yang Yu^1^, Zhenfu Zhang^1^, Yunxin Han^1^, Sha Huang^1^, Jiagui Wu^3,*^, Huan Chen^1,*^, Junbo Yang^1,*^

^1^ College of Science, National University of Defense Technology, Changsha 410073, China

^2^ School of Artificial Intelligence, Chongqing University of Technology, Chongqing 401135, China

^3^ School of Physical Science and Technology Southwest University Chongqing 400715, China

^*^Corresponding author:

[yangjunbo@nudt.edu.cn](mailto:yangjunbo@nudt.edu.cn)

[chenhuan11@nudt.edu.cn](mailto:chenhuan11@nudt.edu.cn)

mgh@swu.edu.cv

S1 Unit cell structure


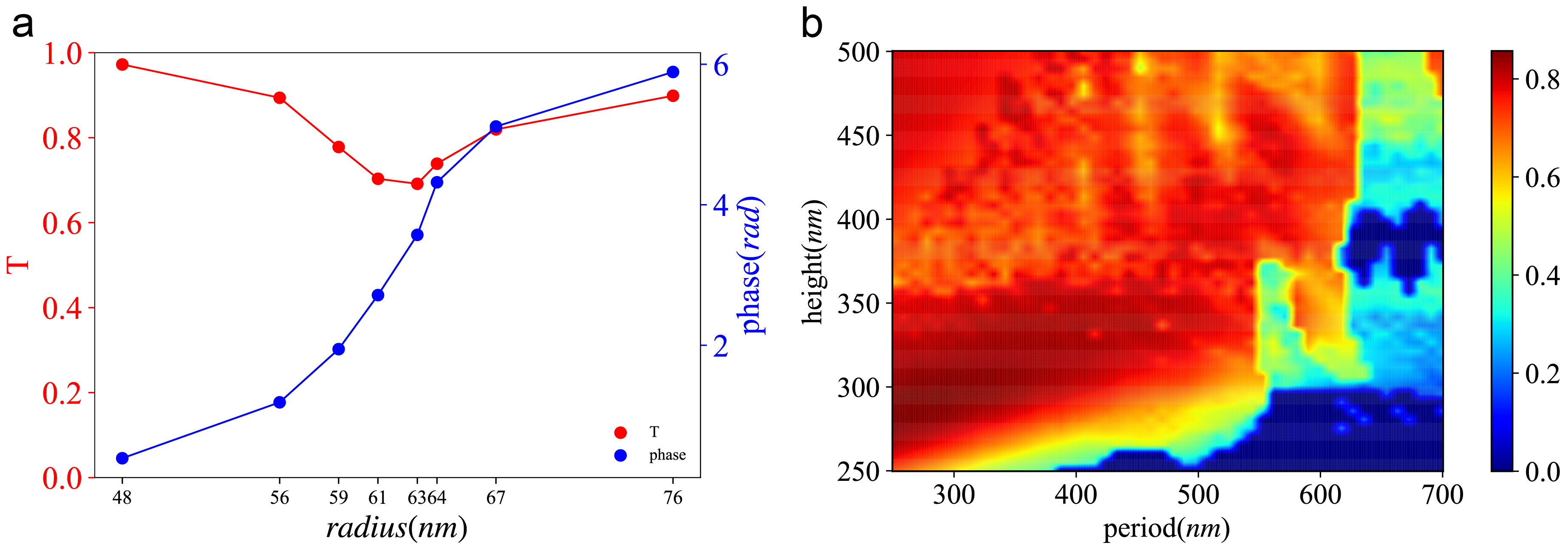


**Figure S1**:(a) The unit cell structure has a period of 347 nm and a height of 378 nm. The red solid line represents the transmittance of the unit cell structure at different radii. The blue solid line represents the phase shift of the unit cell structure at different radii. (b) Transmittance of eight selected unit structures at different heights and periods.

S2 Definition of wavefront error

The ideal focusing phase is calculated using a hyperbolic phase distribution, given by formula (1). The hyperbolic phase distribution enables aberration-free imaging along the axis.[1] To demonstrate the excellent performance of the optimized metalens, we compared the near-field wavefront phase of the metalens with the hyperbolic phase distribution. The closer the metalens wavefront is to the hyperbolic phase, the smaller the overall aberration of the metalens system. Based on this concept, we calculated the phase difference between the metalens wavefront and the hyperbolic phase and then mapped this error to spatial distances to quantify the imaging performance of the metalens system.

$$\begin{aligned} \phi=-\frac{2\pi}{\lambda}\left( \sqrt{x^{2}+y^{2}+f^{2}}-f \right)\#\left( 1 \right) \end{aligned}$$

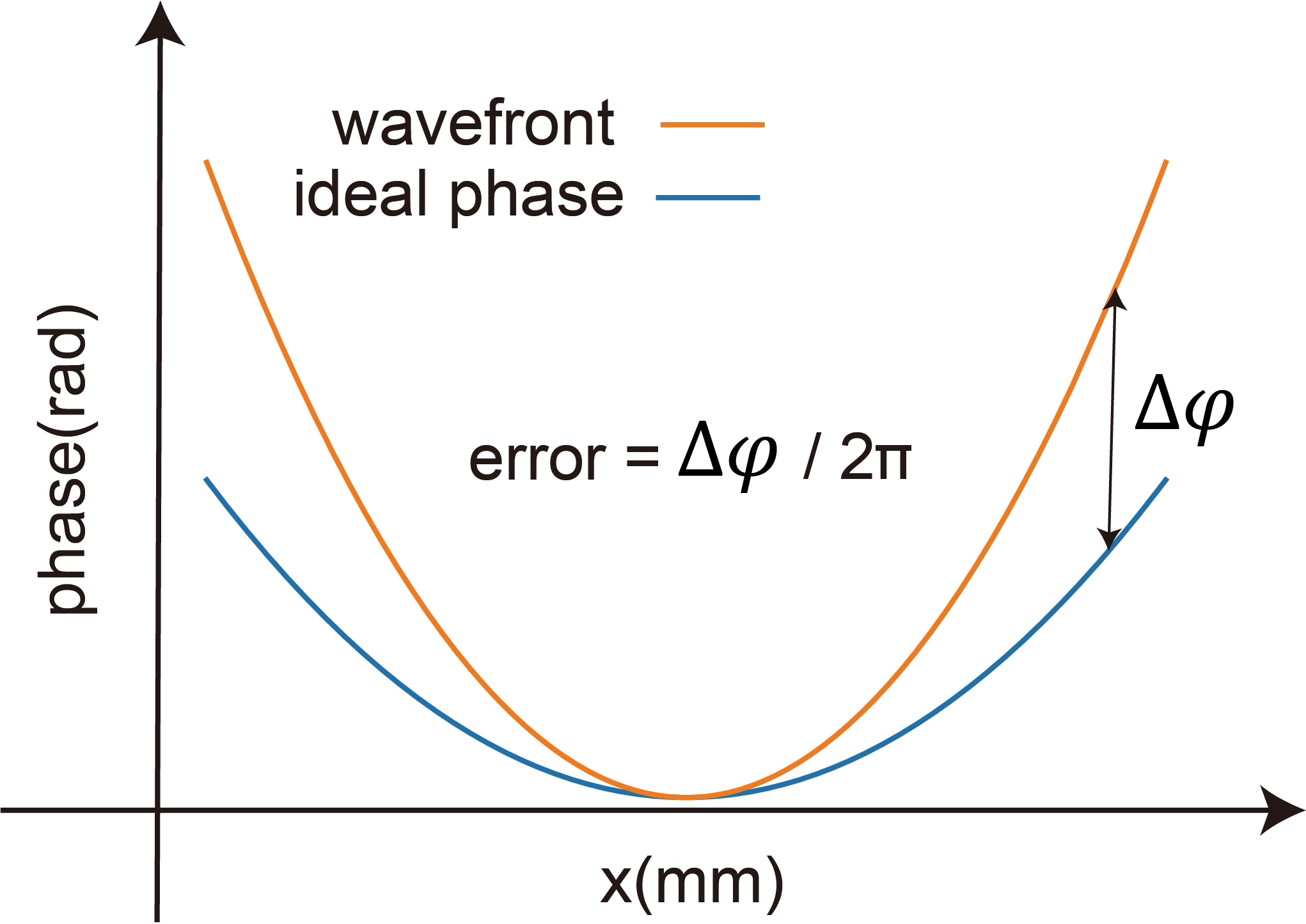


**Figure S2**: The orange solid line represents the wavefront phase of the metalens under test, while the blue solid line represents the ideal focusing wavefront phase. At certain coordinate positions, there is a phase difference between the two. By dividing the phase difference by $2\pi$ the error is mapped to spatial distances.

S3 Experimental setup


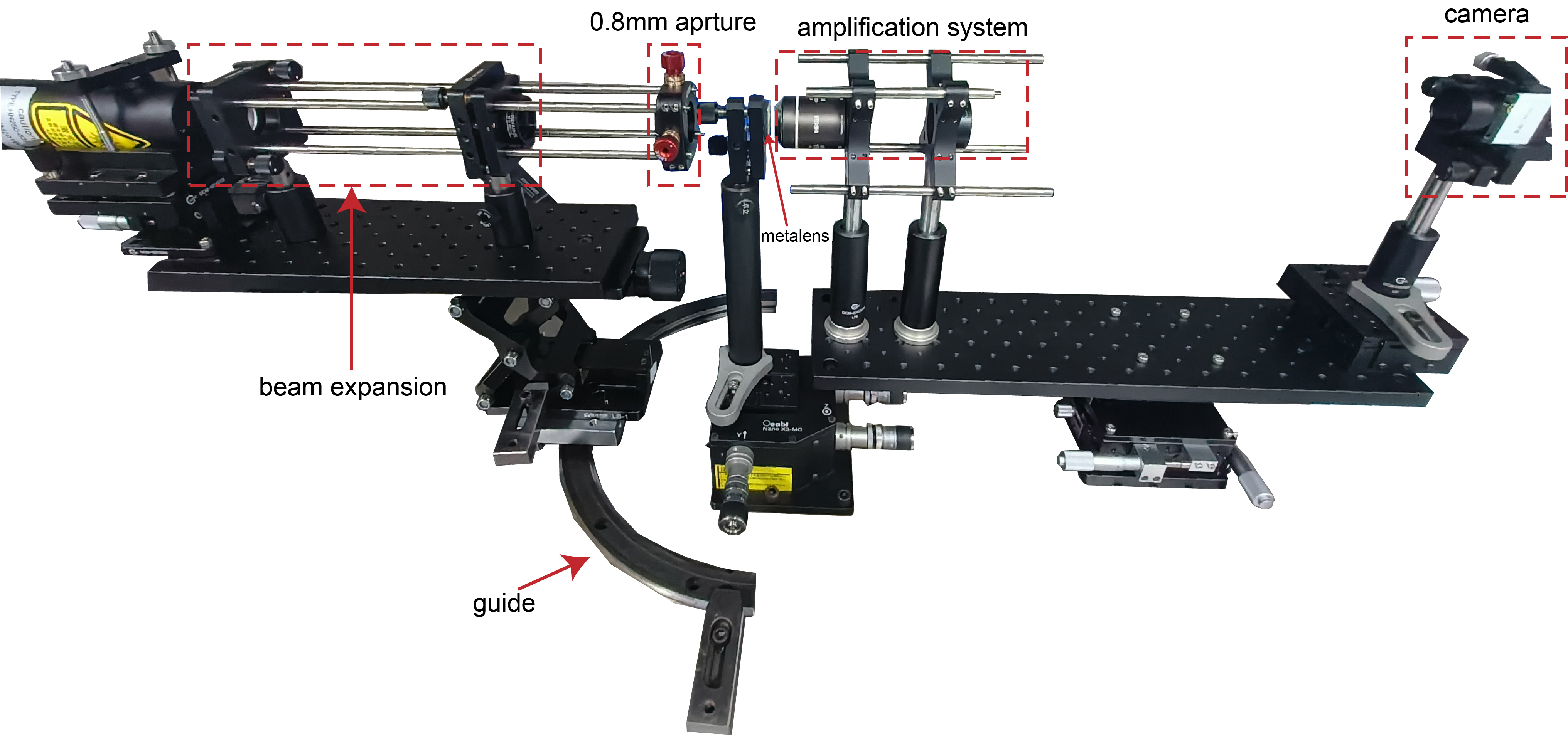


**Figure S3**: Metalens PSF measurement setup. The laser used is the Daheng Optics DH-HN250 with a wavelength of 632.8 nm. The overall system consists of a beam expander, a 0.8 mm aperture stop, the metalens, and an imaging system. The imaging system includes a 100x objective lens and a 300 mm focal length tube lens. The camera used is a monochrome camera with a pixel size of 1.85 µm.

S4 The effect of aperture stop displacement on the PSF (Point Spread Function)

In the main text, we observed that imaging the resolution target still resulted in some edge blurring. There are two main reasons for this. First, the thickness of the aperture stop causes a loss of most frequency information in the edge regions. Second, when the metalens is placed in the aperture stop tube, alignment issues arise due to manufacturing tolerances and manual assembly errors, causing misalignment between the aperture stop and the center of the metalens. Proper alignment is crucial for the imaging quality of the metalens system. Misalignment directly leads to a degradation in imaging quality. However, due to limitations in laboratory conditions, precise alignment of the aperture stop with the metalens is challenging.

To illustrate this effect, we simulated the PSF of the metalens optical system with a misaligned aperture stop. Figure S7a shows a schematic of the aperture stop misalignment. In Figure S7b, the x-axis represents the incident angle, and the y-axis represents the displacement distance of the aperture stop. It is evident that with a 100 µm displacement, the PSF of the metalens system exhibits strong ripples on one side, indicating significant off-axis aberrations. This phenomenon becomes more pronounced with increasing displacement distance. This explains why the integrated metalens camera exhibits slight edge blurring. The primary reason is that the packaging of the metalens prevents the integrated metalens camera from fully realizing the optimized metalens's performance.


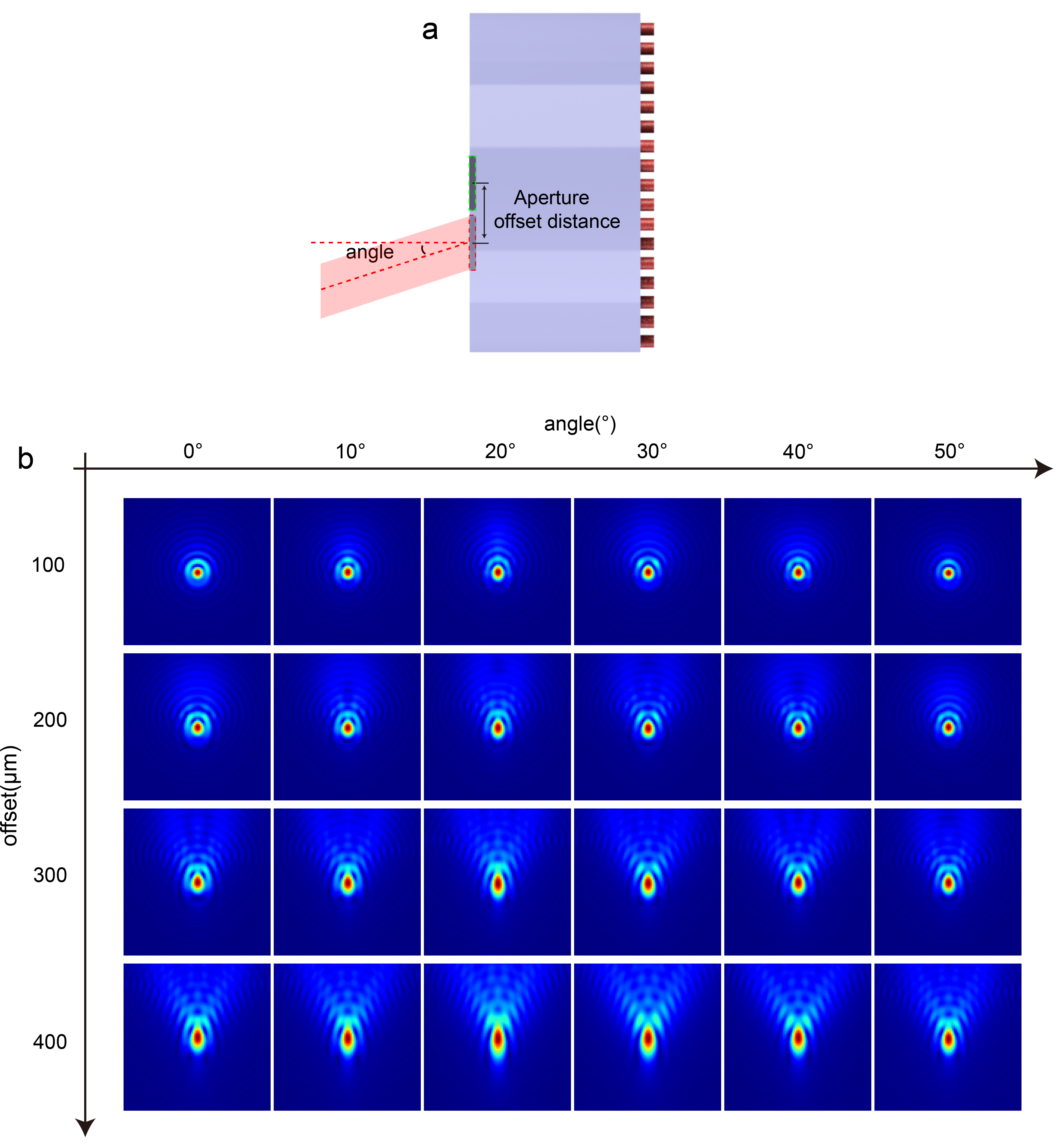


**Figure S4** (a) Diagram illustrating the misalignment of the aperture stop. The aperture stop should ideally be placed within the area outlined by the green dashed circle. However, due to various errors, it is positioned in the area indicated by the red dashed circle. (b) The optimized point spread function (PSF) of the metalens under different incident angles and aperture displacement distances.

S5 The discussion on uneven brightness in captured images


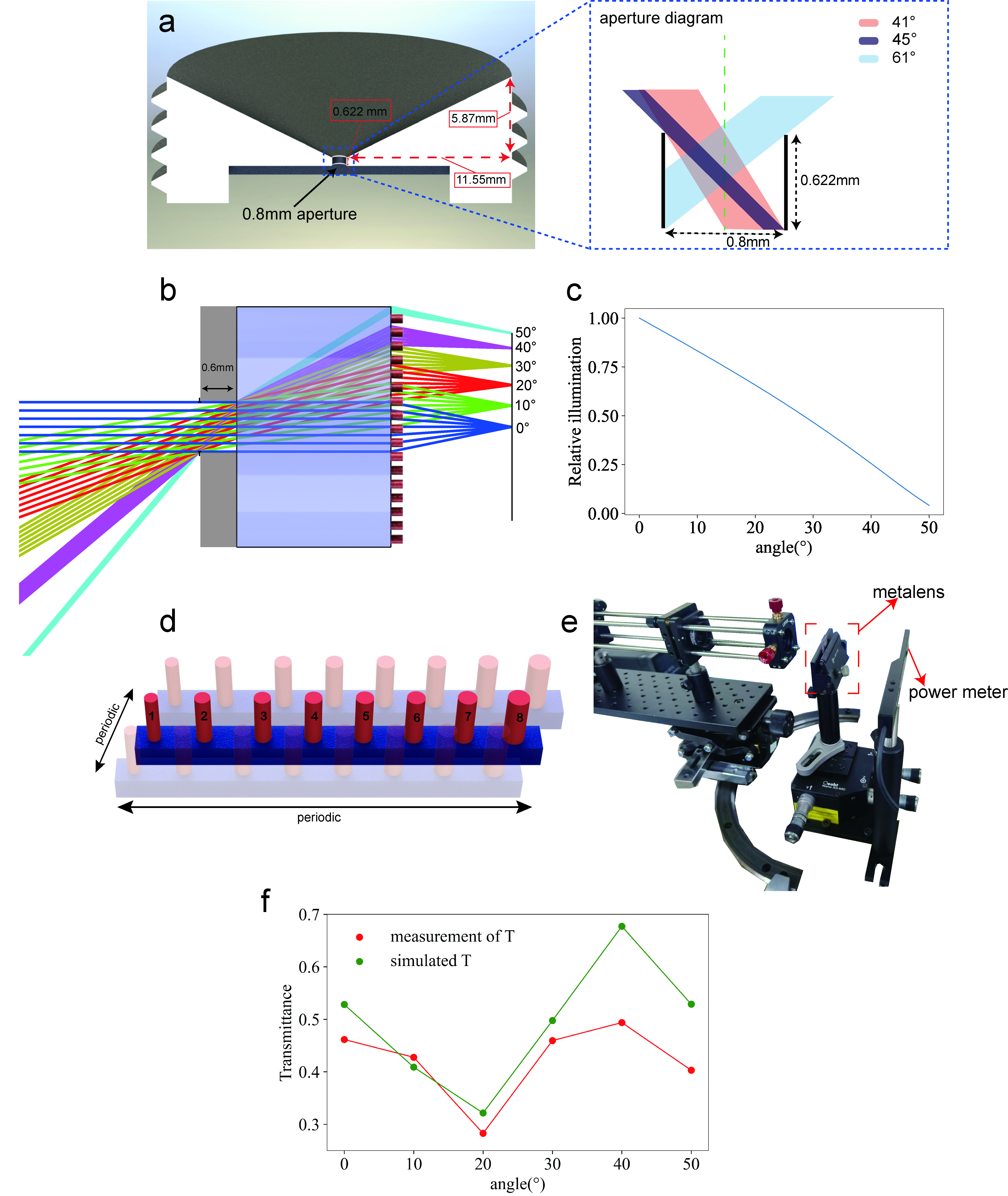


**Figure S5**: (a) Optical tube with an aperture stop for mounting the metalens, with specific dimensions indicated in the figure. The dashed circle on the right illustrates the small aperture. The figure shows the truncation of plane waves incident at angles of 41°, 45°, and 61°. For the 61° incident angle, the light source is completely truncated, indicating that the limit field of view of the aperture stop is 61°. For a 45° incident angle, most of the light is truncated. We consider 45° as the effective observable field of view, and data for this field of view range have been presented in the main text. (b) Schematic diagram of a metalens structure with a vignetting diaphragm. (c) Relative illumination of the image plane at different incident angles. (d) Schematic diagram of the equivalent metasurface structure used in the simulation. (e) Experimental setup for measuring the transmission efficiency of the metalens. (f) Measured transmission efficiency versus simulated transmission efficiency of the metalens in the experiment.

A notable issue observed in the experiment is that the captured images exhibit a brighter center and darker edges. This effect is primarily caused by vignetting due to the thickness of the aperture diaphragm. Our aperture diaphragm has a diameter of 0.8 mm, with a thickness of approximately 0.6 mm due to the thin-wall limitations of 3D printing. A detailed structural diagram is shown in Fig. S5a. The thickness of the aperture diaphragm effectively introduces an unintended vignetting diaphragm at the very front of the metalens system, which partially obstructs light rays from large field-of-view angles, resulting in vignetting, with the phenomenon of a brighter center and darker edges in the image. A schematic diagram of the lens structure with the vignetting diaphragm is shown in Fig. S5b. We calculated the relative illumination on the image plane at different angles, considering the impact of the vignetting diaphragm, based on geometric optics propagation theory, to demonstrate the effect of the aperture diaphragm on imaging uniformity. The detailed data is shown in Fig. S5c. From the figure, it can be seen that as the field angle increases, the relative illumination gradually decreases, dropping to about 4% of the central field at 50°. This is the primary cause of the observed phenomenon of a brighter center and darker edges in the imaging region.

Another important consideration is whether the transmission efficiency of the metalens is also affected by the incident angle. We conducted both numerical simulations and experiments to measure the transmission efficiency of the metalens. In the numerical simulation, due to the large overall volume of the metasurface, it was not feasible to apply FDTD to calculate the entire metalens. Instead, we approximated the overall structure by treating the selected 8 unit cells as a single entity, as shown in Fig. S5(d), assuming that the entire metalens consists of a periodic arrangement of this structure. Periodic boundary conditions were used in the numerical calculations to obtain the simulated transmission efficiency of the metalens. Subsequently, we measured the transmission efficiency at different incident angles using the setup shown in Fig. S5e, with the results presented in Fig. S5f. From the comparison between the experimental and simulated results, it can be seen that the transmission efficiency of the metalens does not vary significantly with the incident angle, especially at larger angles. Therefore, the primary cause of the bright center and dark edges observed in the imaging experiment is not the variation in the metalens transmission efficiency but rather the vignetting effect caused by the vignetting diaphragm at the front of the metalens.

S6 Camera calibration

Our designed large-field near-diffraction-limited metalens exhibit barrel distortion characteristics similar to those found in typical large-field lenses. This is primarily due to the difference between the focal point movement in the ideal imaging model, which follows $ftan\theta,$ and the actual focal point movement, which follows $fsin\theta$. In our experiments, we measured the focal point movement of the optimized metalens by adjusting the magnification system to ensure that the focal spots at different incident angles all fall on the optical axis of the magnification system. This allowed us to determine the focal point movement by measuring the displacement of the magnification system.

The focal point movement following $fsin\theta$ is the main cause of the distortion, but this can be corrected using algorithms. The undistorted images after correction are shown in Figure 5, marked as "Undistorted." During the calibration process, we used an 8×5 inner corner black-and-white checkerboard pattern, capturing multiple images from different angles, as shown in four examples in Figure S8c. We then applied Zhang's calibration technique to calculate the camera's intrinsic parameters and distortion coefficients[2]. With these data, we can use the intrinsic parameters and distortion coefficients to compute the undistorted images.


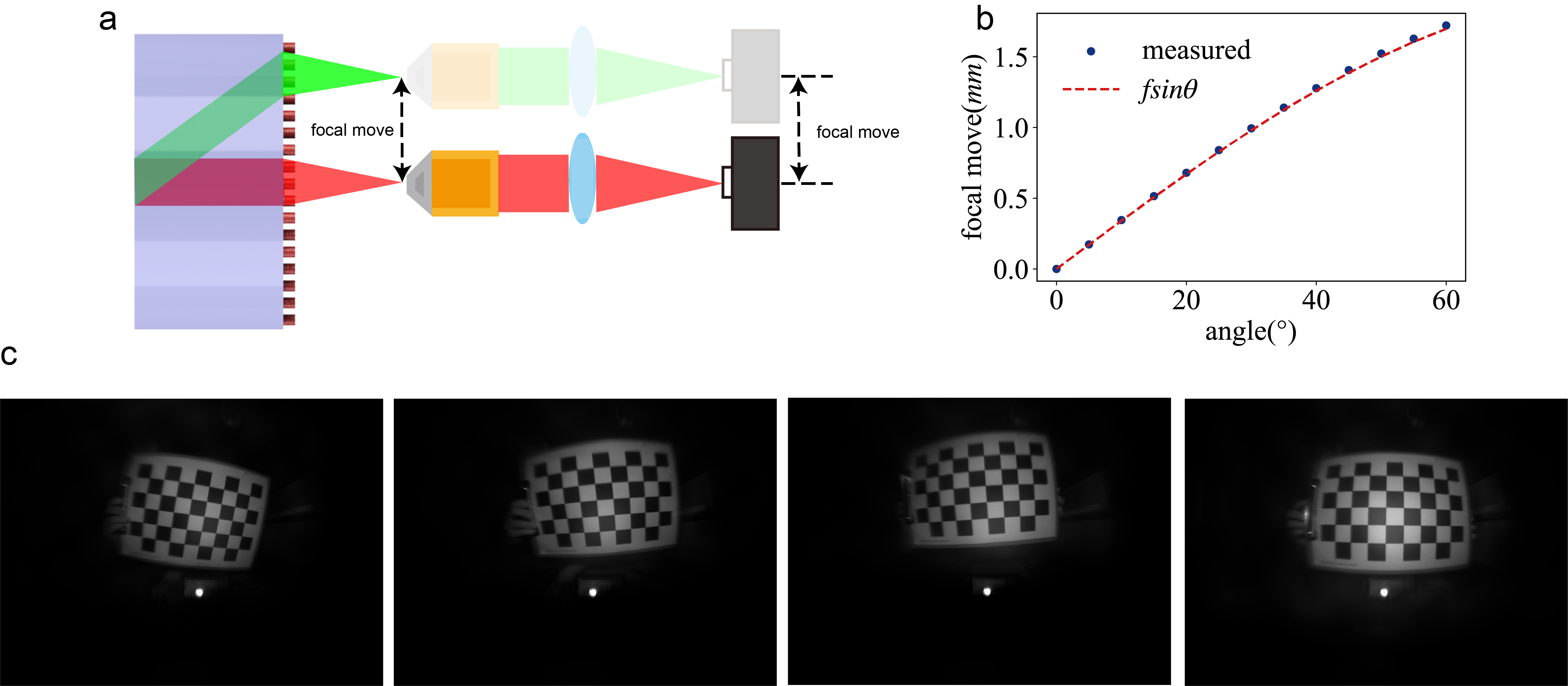


**Figure S6**: (a) The system used for testing focal point movement, is located on the breadboard in the latter part of Figure S6. (b) Measured focal point movement. (c) Capturing photos of the black-and-white checkerboard pattern during the calibration process.

S7 Complete PSF data

In the main text, we presented a partially cropped PSF for clarity. To ensure the accuracy and authenticity of the data, we have provided the complete PSF data.


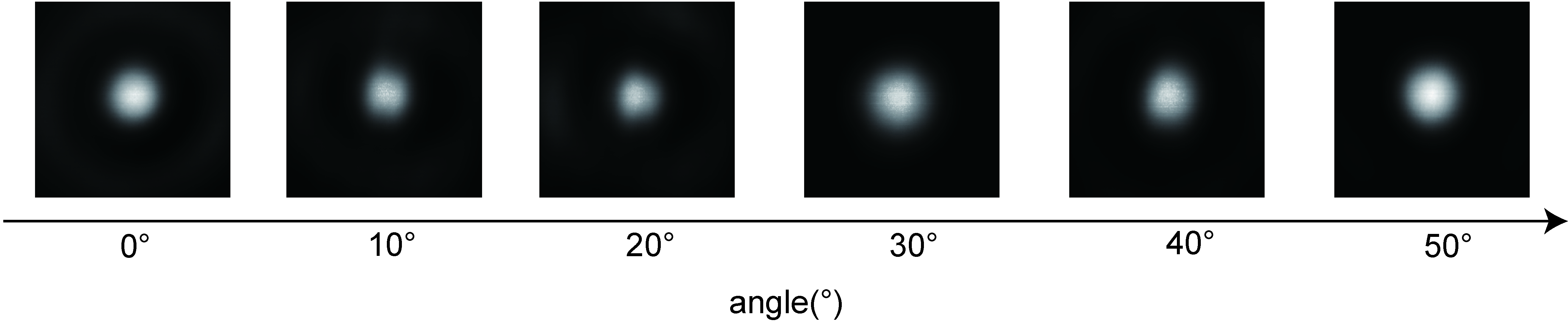


**Figure S7:** The complete PSF data measured in the experiment. References:

REFERENCES

[1] M. Khorasaninejad *et al.*, “Polarization-Insensitive Metalenses at Visible Wavelengths,” *Nano Lett.*, vol. 16, no. 11, Art. no. 11, Nov. 2016, doi: 10.1021/acs.nanolett.6b03626.

[2] Z. Zhang, “A flexible new technique for camera calibration,” *IEEE Trans. Pattern Anal. Machine Intell.*, vol. 22, no. 11, pp. 1330–1334, Nov. 2000, doi: 10.1109/34.888718.
